# Proximal Splenorenal Shunt Surgery for Bleeding Gastric Varices in Non-Cirrhotic Portal Hypertension

**DOI:** 10.7759/cureus.10464

**Published:** 2020-09-15

**Authors:** Utpal Anand, Ramesh Kumar, Rajeev N Priyadarshi, Kunal Parasar, Aaron G John

**Affiliations:** 1 Surgical Gastroenterology, All India Institute of Medical Sciences Patna, Patna, IND; 2 Gastroenterology, All India Institute of Medical Sciences Patna, Patna, IND; 3 Radiology, All India Institute of Medical Sciences Patna, Patna, IND

**Keywords:** proximal splenorenal shunt, non cirrhotic portal hypertension, gastric varices, secondary prophylaxis

## Abstract

Background

The optimal management of gastric variceal bleeding in patients with non-cirrhotic portal hypertension (NCPH) is debatable due to the lack of data from large randomized controlled trials. Here we present our experience on proximal splenorenal shunt (PSRS) surgery in NCPH patients with bleeding gastric varices.

Methods

Over a five-year period, a total of 25 PSRS surgeries were performed and data was collected prospectively. Nineteen extrahepatic portal vein obstruction (EHPVO) and six non-cirrhotic portal fibrosis (NCPF) patients with bleeding fundic or isolated gastric varices and normal liver function were included. The collected data was analyzed retrospectively.

Results

Of the 25 patients who underwent PSRS five were lost to follow-up. Twenty patients (80%) were followed up for a median of 3.4 (1-5) years. Gastric variceal regression was noted in all 20 patients with the disappearance of varices in eight patients. On follow-up, shunt thrombosis was noted in four (20%) patients of whom, two had rebleeding between six months and three years after shunt surgery.

Conclusion

PSRS was effective in controlling gastric variceal hemorrhage in 92% (23 of 25) of patients with preserved liver function.

## Introduction

Non-cirrhotic portal hypertension (NCPH) refers to portal hypertension caused by two vascular diseases of the liver: extrahepatic portal venous obstruction (EHPVO) and non-cirrhotic portal fibrosis (NCPF). Currently, endoscopic sclerotherapy (EST) and endoscopic variceal ligation (EVL) are the standard of care for bleeding esophageal varices (EVs) due to NCPH [1]. The management of gastric varices (GVs) is still evolving [2,3]. The present evidence favors the use of cyanoacrylate glue as first-line therapy for both acute bleeding and secondary prophylactic eradication of gastric varices [4,5]. However, most of the available literature in the management of gastric varices is on cirrhotic patients. Glue injection therapy is technically challenging and is associated with rebleeding rates of 23-50% [6,7]. Also, endoscopic glue often requires multiple sessions which increase the burden of cost, resource utilization, and is unsuitable for patients residing in rural and remote areas. The access to balloon-occluded retrograde transvenous obliteration (BRTO) or transjugular intrahepatic portosystemic shunts (TIPS) for the treatment of gastric varices is often limited in countries with constrained resources [8]. 

There is a paucity of literature regarding the role of surgery in bleeding gastric varices. Splenectomy is the best operation for patients in whom gastric varices are secondary to splenic vein thrombosis and left-sided segmental portal hypertension. There is still no consensus regarding the best surgical option for NCPH and gastric varices in patients with a patent splenic and superior mesenteric vein. There are few reports of patients operated on for bleeding gastric varices undergoing gastric devascularization, under-running of the varices, and splenectomy [9]. However, these techniques were associated with a high incidence of rebleeding [10].

The rebleeding rates of esophageal varices are low in patients of NCPH treated with proximal splenorenal shunts [11]. The role and efficacy of shunt surgery in patients with GV are lacking. The presence of existing spontaneous gastro-renal shunts (GRS) in patients with GV makes the role of shunts controversial. Patients with NCPH have good hepatic function and the risk of death from exsanguinating bleeding is higher than liver failure [11]. Proximal splenorenal shunt (PSRS) surgery in bleeding GVs patients may be an effective single-step therapy for prevention of rebleeding as well as correction of hypersplenism with acceptable morbidity and mortality. Based on the above assumptions, we prospectively treated patients of NCPH who had bled from gastric varices with PSRS as secondary prophylaxis and herewith present our experience. 

## Materials and methods

**Figure 1:**
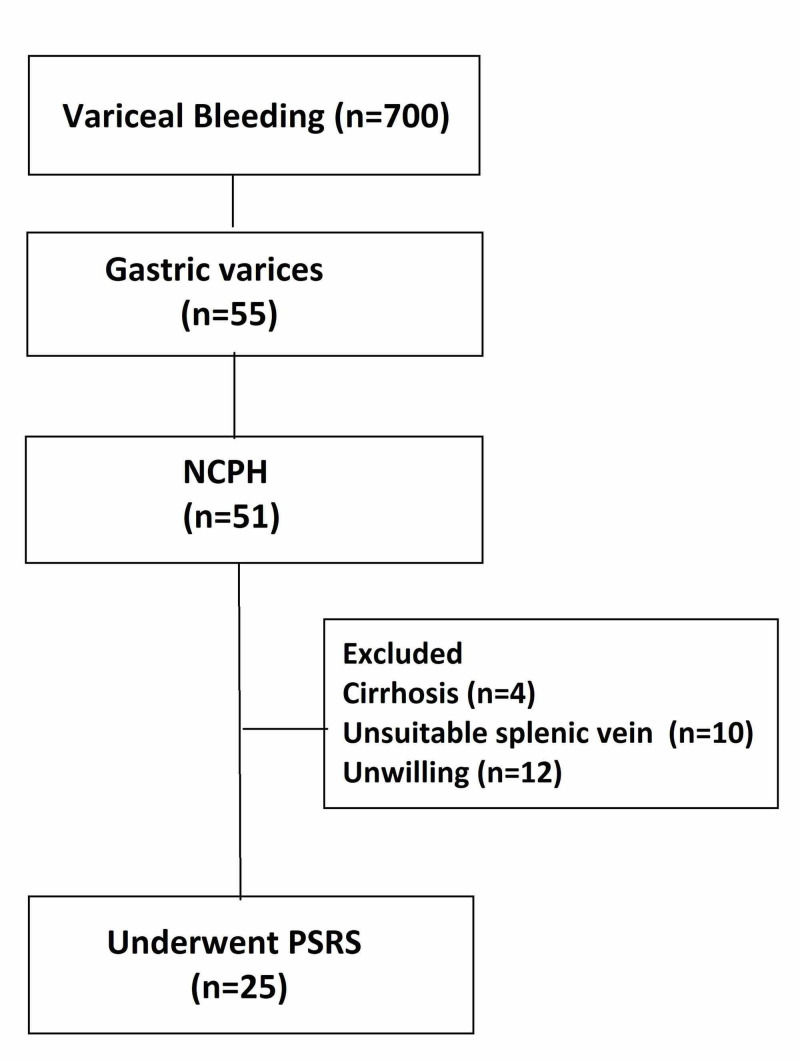
Flowchart of patient selection NCPH - Non-cirrhotic portal hypertension; PSRS - Proximal splenorenal shunt

Between January 2013 and June 2018, a total of 700 patients presented to the gastroenterology service with variceal bleeding. Of these, 55 patients had gastric varices; 51 of them were non-cirrhotic (NCPH). After initial endoscopic therapy with glue injection; patients who failed endoscopic therapy, had symptomatic hypersplenism, or those residing in remote places with no access to endoscopic treatment were considered for PSRS surgery. After excluding four patients with suspicion of underlying cirrhosis, 10 patients with unsuitable splenic vein on doppler ultrasound, and 12 patients unwilling for surgery, 25 patients (19 EHPVO and six NCPF) underwent PSRS (Figure 1).

All patients underwent a thorough clinical history, physical examination, and relevant blood investigations at the initial visit. Doppler ultrasonography (USG) and upper GI endoscopy (UGIE) were done to evaluate the portal system and varices in all patients. Patients were subjected to PSRS surgery on an elective or a semi-elective basis. All patients underwent PSRS by thoracoabdominal incision. A thoracoabdominal incision was used for better exposure and minimal requirement of sophisticated energy sources. Anastomosis between the splenic vein and left renal vein was done with 6-0 prolene. Heparin (100 units/kg) was administered during surgery after mobilization of the splenic vein and before clamping the left renal vein. In addition to the shunt procedure, the disconnection of large varices between the fundus of the stomach and retroperitoneum was also done in all patients. Liver biopsy was taken intraoperatively in all patients.

**Figure 2:**
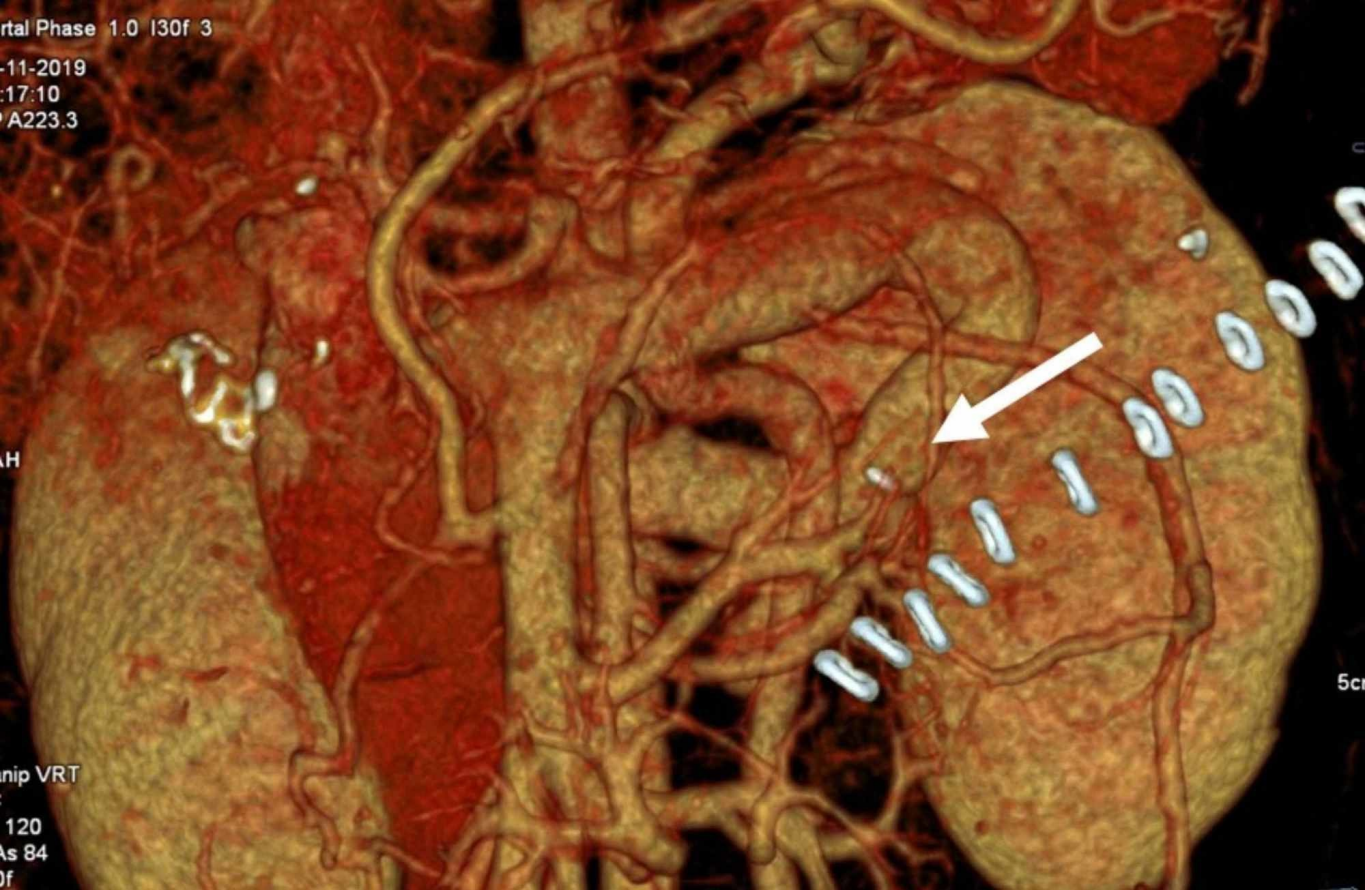
The volume rendering multidetector CT image in the portal venous phase shows the opacified patent surgically created shunt vessels communicating with the left renal vein.

Data on the duration of surgery, length of hospital stay, correction of associated hypersplenism, post-operative complications, rebleeding, and mortality was collected prospectively. Patients were followed up every three months for one year, every six months for the second year, and yearly thereafter with routine outpatient visits, and telephonically if unable to come for a routine visit. The shunt was considered patent when the vessel lumen opacified with contrast to be clearly identified on volume rendering 3D image (Figure 2) or when the Doppler USG depicted blood flow across the shunt vessel. Standard methods were used for data analysis using SPSS version 22 Statistical analysis software.

## Results

Twenty-five NCPH patients were included: 19 patients with EHPVO and six patients with NCPF. The mean (± SD) age at presentation was 18.6 ± 3 years (range, 12 - 34 years) and the majority of the patients were male (19/25, 76%). The median number of bleeding episodes prior to surgery was two (1 - 6) (Table 1).

**Table 1:** Patient demographics EHPVO - Extrahepatic portal vein obstruction; NCPF - Non-cirrhotic portal fibrosis

Characteristic	n=25
Age mean (range)	18.6 (12 - 34)
Sex	
Male (%)	19 (76)
Female (%)	6 (24)
Number of bleeding episodes prior to surgery (range)	2 (1 – 6)
Diagnosis	
EHPVO	19
NCPF	6

All patients had normal liver functions and varying degrees of cytopenia suggesting hypersplenism. An end-to-side PSRS was done in all patients. The mean shunt diameter was 8.4 mm (range 4.5 - 10.5; SD 3.4 mm). The average operative time was 4.5 ± 0.5 hours and the average blood loss during the operation was 865 ± 315mL. All patients underwent a splenectomy and the median (range) weight of detached spleen was 1100 (660-1260) grams. The patients were discharged after stays of 6 ± 2 days after surgery (Table 2). 

**Table 2:** Operative details and postoperative follow-up

Parameter	n=25
Shunt diameter mean in mm (range)	8.4 ± 3.4
Operative time in hours	4.5 ± 0.5
Blood loss in mL	865 ± 315
Spleen size in grams (range)	1100 (660-1260)
Length of stay in days	6 ± 2
Follow-up	n=20
Variceal regression	20
Complete variceal regression	8
Shunt thrombosis	4
Variceal rebleeding	2
Hepatic encephalopathy	0

None of the 25 patients died during follow-up. There were two episodes of early rebleeding within 48 hours of operation. CT angiography was performed in both patients, demonstrating a blocked shunt and extensive thrombosis of the splenic vein and superior mesenteric vein in one patient. Both patients responded to conservative treatment with transfusion of blood and fresh frozen plasma. Both patients were kept for endoscopic follow-up and had no evidence of rebleeding. In addition to the above, another patient with NCPF presented with weakness and passage of black tarry stool without hematemesis. Doppler ultrasonography showed that the shunt was patent. Upper GI endoscopy showed duodenal ulcer bleed which was managed by argon plasma coagulation. One patient with NCPF had post-operative hepatic decompensation and developed ascites. He responded well to medical treatment and had no further episodes of bleeding or hepatic decompensation on follow up. Hypersplenism, characterized by anemia, leucopenia, and thrombocytopenia with normal or hypercellular bone marrow, was present in all 25 patients, which reversed after shunt surgery.

Twenty patients (80%) were followed up for a median period of 3.4 years (range 1-5 years). Gastric varices regressed in all the 20 patients with patent shunts with complete disappearance of gastric varices in eight patients. Five patients were lost to follow-up after a six-month period. During follow-up, shunt thrombosis was noted in 20% (4 of 20) of patients and two of them developed rebleeding six months and three years, respectively, after shunt surgery. None of the patients developed encephalopathy during follow-up.

## Discussion

Our experience indicates that PSRS as a secondary prophylaxis in NCPH patients with a history of bleeding gastric varices is an effective and safe procedure. Although the shunt thrombosis occurred in 20% of the patients during the mean follow-up period of 3.4 years, the rebleeding rate was only 10% and was successfully managed by endoscopic therapy.

The GVs are relatively larger in size and are associated with natural shunts, resulting in lower portal pressures in comparison to EVs [12]. Even a small rise in portal pressure increases the wall stress many times due to larger radius of GVs which results in their rupture [13,14]. Therefore, the methods used to control the bleeding in EVs are technically difficult to practice for GVs. There are no head-to-head studies comparing endoscopic and surgical therapy for controlling and preventing bleeding in gastric varices. Conventionally, the first-line method in managing the gastric varices is endoscopic variceal obliteration (EVO) using N-butyl-2-cyanoacrylate glue [1,2] with a rebleeding rate between 22% and 37%. Shunt surgery is not the standard of care in developed nations where TIPS and endoscopic capabilities are readily available.

Long-term studies (15-40 years) from India and other countries have shown that shunt surgery with or without splenectomy (proximal splenorenal, side-to-side splenorenal, distal splenorenal, or mesocaval) is associated with shunt patency rates between 90% and 95%, rebleeding rates between 5% and 10%, absence of portosystemic encephalopathy, and 15-year actual survival rates of 95% for NCPH patients with esophageal variceal bleed [15,16]. There is limited data in the literature defining the role of shunt surgery in NCPH and GVs bleeding. Thomas et al [17] studied distal splenorenal shunt (Warren shunt) in 30 patients with bleeding gastric varices and preserved liver function where six patients had cirrhosis, four had NCPF, and 20 had EHPVO. In 26 patients, gastric varices disappeared and concomitant esophageal varices showed marked regression with no bleeding at follow-up of 21 months.

In our experience, the role of PSRS in controlling GVs rebleeding is comparable to other reported series with longer follow-up periods. We found PSRS more suitable in NCPH patients due to the associated massive splenomegaly causing discomfort, pain, and hypersplenism, which is not reversed completely after distal splenorenal shunts.

EVO requires 4 -10 sessions over 8 - 20 weeks for complete obliteration of varices [18]. In our experience, there is a significant drop out rate of patients on glue therapy due to financial constraints and poor accessibility for patients from remote places. PSRS, being a single step procedure with minimal morbidity, can be a better alternative for these patients.

Furthermore, EVO has many technical difficulties (para-variceal injection, needle sticking in varix, intra-peritoneal injection leading to peritonitis and adherence of the glue to the endoscope) or complications (fever, paravariceal injection with mucosal necrosis and bleeding, embolization into the renal vein, IVC, pulmonary or systemic vessels and retro-gastric abscesses) [19]. Although post-shunt encephalopathy, myelopathy, and nephropathy have been reported from various series in NCPH, mostly NCPF, our patients did not develop any such complications untill the last follow-up [20]. There is no report of post-splenectomy sepsis in any of these patients to date.

## Conclusions

In our study of the 20 patients on follow-up, all had variceal regression, which was complete in eight, and none of them developed any major complications. This suggests that PSRS is safe and effective in preventing gastric variceal rebleeding in patients with EHPVO or NCPF. PSRS is a one-time solution. It has the added advantage of reversing the changes of hypersplenism.

Considering the limitations of endoscopic glue therapy, PSRS can be considered a viable alternative in this group of patients. An obvious limitation of our study is the small sample size and lack of a control arm.
